# Assessment of frontal lobe functions in a sample of male cannabis users currently in abstinence: correlations with duration of use and their functional outcomes

**DOI:** 10.1186/s42238-024-00244-w

**Published:** 2024-08-21

**Authors:** El-Shimaa Tag-Eldeen, Magda Fahmy, Khaled Anwar, Omneya Ibrahim

**Affiliations:** Department of Psychiatry and Neurology, Suez Canal University, Ismailia, Egypt

**Keywords:** Chronic cannabis use, Duration of use, Abstinence, Frontal lobe assessment battery, General assessment of function

## Abstract

**Background:**

Previous research literature reported different results regarding the long-term effects that cannabis use can exert on the frontal lobe neurocognitive functions of its users. Another body of research suggested that cannabis use negatively affects the person’s general level of occupational and psychosocial functioning consequently to these alterations. Some other research results did not support these findings. To date, it is still debatable whether chronic cannabis use triggers negative neurocognitive effects in chronic users even after a period of abstinence. Research data exploring consequent adverse outcomes on the general individual occupational and psychosocial functioning is not yet conclusive.

**Results:**

We conducted this study to examine the residual neurocognitive effects of cannabis use, whether it is affected by duration of cannabis use before abstinence, and its relation to individual’s global assessment of functioning exhibited in the person’s occupational and social life whether it’s family or friends. Our sample comprised 80 male participants (18–45 years old) who were grouped into 4 groups (3 groups with different durations of use and a control group), with no significant difference between the four studied groups regarding age, education, and socioeconomic level. The Kruskal Wallis test was used to test the significance of differences in the distribution of total frontal lobe battery results and the general assessment of function scores using GAF scores between study groups. Post hoc testing was performed to adjust for multiple comparisons using Bonferroni method.

**Conclusion:**

Data analysis showed that cannabis users experienced general functional disturbances that encompass impairments in social and occupational life aspects. These impairments in function are correlated with the presence of neurocognitive deficits even after a period of abstinence. Both having significant positive correlation with longer duration of cannabis use.

**Supplementary Information:**

The online version contains supplementary material available at 10.1186/s42238-024-00244-w.

## Background

Cannabis use is a current global health concern. In 2020, the WHO estimated that there were 147 million consumers of cannabis, equivalent to 2.5% of the world’s population, with Cannabis Use Disorder (CUD) cases reaching 23.8 million CUDs (WHO | Cannabis 2020). In a systematic analysis, an estimated figure of 646,000 years of healthy life lost to disability was reported as a consequence of cannabis use (Degenhardt et al. 2018).

Cannabis use disorder defined as a chronic, relapsing condition, with a core feature of loss of control over cannabis use. This loss of control is reflected in persistent use of cannabis despite adverse consequences (American Psychiatric Association 2022).

Cannabis use yields different health effects on users. More attention has been increasingly given recently to cannabis effects on the brain. Cannabis action within the central nervous system is via indirect neurotransmission modulation (Brunt and Bossong 2022), through the endocannabinoid system that is present throughout the entire brain, with CB1 (cannabinoid receptors 1) located throughout the cortex, including the frontal lobe and prefrontal cortex (Mackie 2008; Zou and Kumar 2018).

Frontal lobes are paramount to accomplishing human’s higher cognitive functions, with the prefrontal cortex (PFC) being at the summit of this hierarchal, timely sophisticated interplay, integrating the cognitive representations of various perceptual and other inputs towards conceptualizing and accomplishing goal-directed behaviors (Fuster 2001; Alexander and Brown 2018; Haber et al, 2022). This entails sophisticated, successive decision making processes that are crucial for successfully and appropriately navigating through the never-ending subtle social interactions encountered throughout everyday life (Firat 2019).

Accumulated research data from a recent body of research presented evidence of existing association between poorer neurocognitive performance and the current use or acute intoxication of cannabis (Oomen et al. 2018; Bourque and Potvin 2021; Ramaekers et al. 2021). Cannabis was found to come in the third place within the list of the substances that impair workplace productivity, with attributable productivity losses linked to presenteeism or absenteeism along with premature mortality and long-term disability (Sorge et al. 2020). Among the notable effects on the central nervous system linked to the mentioned impairments are the lack of concentration, drowsiness, thought formation and expression errors, sedation along with impaired learning and memory (Phillips et al. 2015). It is also evident that driving under the influence of cannabis increases the risk of traffic collisions (Howard and Osborne 2020). Cannabis users have significantly slower and less efficient performance on psychomotor speed and visuomotor processing tests. This can be related to greater glucocorticoid exposure in the prefrontal cortex due to cumulative effects of cannabis use. Prolonged glucocorticoid exposure damages the prefrontal cortical function. This can lead to altered inhibitory regulation of the Hypothalamic Pituitary Adrenal axis (King et al. 2011).

Chronic THC administration leads to CB1 receptors downregulation (Dlugos et al. 2012) and thus blunted cortisol reactivity in response to stresses is typically shown in chronic cannabis users (Cuttler et al. 2017; Al’Absi and Allen, 2021; Glodosky et al. 2021). In normal individuals, dorsolateral PFC was found to undergo increased activity during the exposure to psychosocial stress situations occurring simultaneously with increase in both subjective stress levels and salivary cortisol concentrations, supposedly enhancing coping with the ongoing stressor (Meier and Schwabe 2024). Cortisol release, either within its daily circadian rhythm or in response to stressors, has notable effects on the cognitive and behavioral performances of the person (James et al. 2023). Chronic cannabis use was found to disrupt this rhythm and was associated with blunted positive and negative affective responses to acute stress, and this indicated the presence of emotional dysregulation in this population (DeAngelis and al’Absi 2020).

In addition to the existing evidence that chronic cannabis use has effects on overall health in general and neurocognitive functions in particular, cannabis is further known to have a wide range of ramifications on an individual’s behavior and psychosocial function as well as one’s occupational functions. Propensity for adverse behavioral and functional consequences have been widely reported to arise from cannabis use (Sorkhou et al. 2021).

More research attention has been given in the past few years to explore the evidence of enduring neurocognitive deficits of cannabis use (Lubman et al. 2015; Duperrouzel et al. 2020; Figueiredo et al. 2020; Klugah-Brown et al. 2020; Crocker et al. 2021; Colyer-Patel et al. 2024). A basis for such a rise in research interest in this particular area of cannabis research could be attributed partly to the notable upsurge in the prevalence of use (United Nations Office on Drugs and Crime 2021) along with the accompanying decline in risk perception (Baral et al. 2024) and probably also to the current contrasting findings as well (Macedo et al. 2024; Scott et al. 2018). Thus, it is still challenging to clearly outline the magnitude of the lasting negative effects on brain neurocognition and functions in a person’s lifetime.

As presented earlier, a body of current data suggests the association of chronic cannabis use and the neurocognitive functions of an individual, assessed through standardized neuropsychological tests. To a lesser extent, some others reported these results to be valid even after a period of abstinence, mostly through the same kind of assessments. To date, these research findings are still debatable. Few existing studies have explored the reflection of these findings on everyday function of the individual chronic cannabis user. Such evidence, if present, can be essential in the person’s informed decision of starting, continuing, abstaining from, or returning to cannabis use. This holds truth specifically in communities where using the scientific data related to neuropsychological changes only is not comprehensible or sufficiently convincing.

We conducted this research to explore the potential association between Cannabis Use Disorder (CUD) and the neurocognitive frontal lobe (higher) functions using standardized differential tasks to quantify the level of cognitive impairment. Furthermore, determine the associated (if any) impact on the everyday occupational and psychosocial functioning level of the normal individual compared to a group of non-users. Our study focused on adult men (more than 18 years old) with a clearly delineated period of abstinence prior the day of interviewing and testing.

In this study, we hypothesized that CUD (currently in abstinence) group have neurocognitive deficits upon tested through the Frontal Lobe Function assessment battery and that there is an association between these performances and the duration and age of onset of the cannabis use. We expected also to find an association between these findings and the occupational and psychosocial general functioning levels assessed through the General Assessment of Function (GAF) scale, compared to the non-users control group.

Thus, the following research questions were examined:

1. Are frontal lobe neurocognitive functions assessed through the Frontal Lobe Battery (FAB) affected in Cannabis Use Disorder (CUD) adult men abstinent for a period of more than one month in comparison to a matched control group of non-users?
2. Is there an association between the level of the neurocognitive functioning in the mentioned above sample and both the age of onset and the duration of cannabis use compared to the control group?
3. Is there an association between the occupational and psychosocial general functioning levels assessed through the General Assessment of Function (GAF) scale in the mentioned above sample and the duration of cannabis use compared to the control group?
4. Is there any relation between frontal lobe neurocognitive functions in the studied abstinent CUD sample and their level of occupational and psychosocial general functioning assessed on the General Assessment of Function (GAF) scale when compared to the results on the same scale for the control group?

## Methods

### Design of the study

This is a Cross- sectional (case- control) observational study.

### Participants and procedures

The study was carried out at outpatient clinics of the National Center of Addiction Treatment in Ismailia city, Egypt.

### Participants

Cases recruited were treatment-seeking males who were primary cannabis users, fulfilling the diagnostic criteria for DSM-5 Cannabis Use Disorder (CUD). The abstinence period that was set as a condition for inclusion was one to three months. Age inclusion was between eighteen and forty-five years old who has completed their middle education. Volunteer males among the relatives accompanying the participating cases, with matched age, socioeconomic status, and educational level, and who did not meet the DSM-5 criteria for alcohol/substance abuse or dependence with no lifetime exposure to cannabis were included as the control group. They were included after taking a full medical and psychiatric history and performing clinical assessments to exclude any previous or current history of substance abuse. Urine analysis (for drug screening) was obtained as well before participation to exclude cannabis and any other current substance use disorder.

We did not include within the participants in our study any subjects with history of any neurological condition that could affect the frontal cognitive functions (epilepsy, cerebrovascular strokes, multiple sclerosis, history of a traumatic head injury/unconsciousness) or comorbidity with an intellectual disability or autism spectrum disorder or any general medical condition or psychiatric disorder. For the users group we excluded polysubstance users or those presenting with any withdrawal symptoms except craving. Positive urine screening for cannabis or any other psychoactive substances in both groups was an exclusion as well. All participants of all groups were falling within the average or above average intelligence range by a neuropsychologist.

### Sample size

Using the ANOVA procedure (F test) in the G*Power software (version 3.1.9.6), a total sample size of 76 was large enough to detect an expected effect size (f) of 0.40 (according to Cohen’s criteria) between the four study groups, at 0.05 alpha error probability and 0.80 power. Accordingly, an equal sample of 20 per group was predetermined for this study.

### Procedure

The Ethics and Clinical Research Committee of the Faculty of Medicine, Suez Canal University, (located in Ismailia, Egypt) approved the study protocol before the commencement of any further study procedures.

An informed consent explaining in detail the study design and aim prior to enrollment was introduced to participants before obtaining their written consent.

Participants were free to terminate their participation at any time they desired without any consequences on their treatment or any other rights. All measures to ensure confidentiality of participants’ data were taken by the study principal investigator. We informed the participants that the results of this study could be used for scientific publication without the disclosure of any of their personal details.

Participants accepting to take part in the study underwent investigations and interviews that were not normally a part of their treatment interviews. Testing was conducted in a quiet room. The average time for each interview session for the study related assessments was about three hours.

## Measures and instruments

All participants were subjected to the following:

### History and clinical assessment

- Full history taking includes socio-demographic data in a predesigned questionnaire, which included age, marital status, residency, employment status, income, education, and occupation. Details of cannabis use disorder for users were obtained including the age at onset of use and duration of use.
- Full medical and surgical history was obtained from all participants. Mental status examination was done in addition to full medical examination (including full neurological examination) was conducted for all included participants followed by administration of the cognitive measures for those who fulfilled the inclusion criteria. Upon completion of the study, all participants were provided with debriefing.
- Urine analysis for substance was done to exclude polysubstance use and confirm the duration of abstinence from cannabis.
- Duration of cannabis use was measured by asking respondents how many years they have been using cannabis. Duration responses were categorical. Subjects were then divided into three groups and coded according to their duration of use as follows: group (1) was the control, group (2)1–2 years, (3) 5–6 years, (4) 9–10 years, participants were categorized according to the duration ranges that presented to the clinic and that were eligible for inclusion within our patient selection criteria.
- Then all participants were interviewed using the following tools:

### Structured clinical interview for DSM-5 – Research Version (SCID-5 - RV)

We used the Structured Clinical Interview for DSM 5 (SCID 5) (First 2015) in this study to confirm Cannabis Use Disorder (CUD) diagnosis and to exclude other comorbid psychiatric disorders.

### The Frontal Assessment Battery (FAB)

It was devised as a screening of frontal lobe functions. It includes six subtests (Conceptualization, Verbal Fluency, Motor Programming, Conflicting Instructions, Motor Inhibitory Control, and Prehension Behavior) each evaluating an aspect of frontal lobe function as abstract reasoning, mental flexibility, motor programming and executive control of action, resistance to interference, conceptualization, inhibitory control, self-regulation, and environmental autonomy. It is a simple, applicable, and conclusive battery with overall needed time around 10 min (Dubois et al. 2000; Ichikawa 2011).

The overall performance on these subtests gives a composite score that evaluates the extent of severity of the dysfunction. Scoring on the Frontal Assessment Battery has a distinct graded value for each subtest performance e.g., Conceptualization and abstraction (that is assessed by recognizing similarities) would be with graded as a standardized score of 3 for 3 correct answers, 2 of 2 correct answers, 1 for only one correct answer, 0 for none correct answers. Another example is “Motor programming” (assessed by motor series, as “fist–palm–edge,”, both with examiner and alone). The participant will score 3 if successfully performed six consecutive series alone, score 2 if performed more than 3 series alone, score 1 for 3 successful series but with the examiner and 0 was given to participants who failed to perform 3 successful series whether alone or with the examiner.

### The Global Assessment of functioning scale (GAF) scale

Functional status was the outcome measure we were investigating. This was evaluated on the Global Assessment of Functioning scale (GAF) scale. This scale is used by mental health clinicians and physicians to assess; in a subjective manner, how participants were severely affected regarding social, psychological, and occupational aspects of adults’ functioning on a numeric scale (1–100). It was first described by Hall et al., 1995 and included within the DSM-IV-TR. (Hall 1995; American Psychiatric Association 2000).

### Analysis of data

The calculated sample size of study participants was 80, 20 for each group. The collected data were computerized and statistically analyzed using SPSS program (Statistical Package for Social Science) version 26. Data was tested for normal distribution using the Shapiro Walk test. The Kruskal Wallis test was used to test the significance of differences in the distribution of total FAB and GAF scores between study groups. Post hoc testing was performed to adjust for multiple comparisons using Bonferroni method. Partial correlations were used to investigate the adjusted correlations between the duration of Cannabis use, FAB, GAF, and age of onset. Associations between the study groups and domain-specific FAB variables were tested with Fisher-Freeman-Halton Exact Test since many cells had zero values and more than 20% of cells had expected values less than 5. Quantile regression analysis (for median value) was performed to adjust for the confounding effect of age of onset on the association between the duration of Cannabis use and FAB and GAF scores. Parameters estimates were presented as coefficients, standard errors, 95% confidence intervals. P-values less than 0.05 were considered statistically significant at 95% level of confidence.

## Results

Our study was done at the National Center of Addiction located in the city of Ismailia, one of the eastern governorates in Egypt. The sample was recruited from 2020 till 2022. The aim of this study was to assess whether chronic cannabis use in CUD patients poses any neurocognitive effects even after a minimal abstinence period of one month. Chronic use was categorized into different durations. We assessed the presence of any association of the neurocognitive (the frontal lobe function assessment battery) results with the assessment of the person’s current and social and occupational functions (Global Assessment of Functioning scale).

This study included a control group (Group I), and three groups of different Cannabis use duration (II: 1–2 years; III: 5–6 years; IV: 9–10 years). All groups show comparable socioeconomic characteristics with no significant differences regarding age, education, and socioeconomic level. We studied the distribution of age of onset throughout the whole sample and presented the analysis in a histogram as a supplement file 1 (S1). We also analyzed the distribution of cannabis use duration across the whole sample and provided it as a supplement file 2 (S2).

Table 1 shows that the four groups under study were comparable with no significant difference in terms of age, socioeconomic status, education, employment, and marital status.

**Table 1:** Sociodemographic data of studied groups

Variable		Group I *n* = 20	Group II *n* = 20	Group III *n* = 20	Group IV *n* = 20	*P* value
Age (years)	Mean ± SD	24.3 ± 5.1	23.5 ± 2.3	24.9 ± 3.9	25.1 ± 2.1	0.160a
Socioeconomic level	Low	7 (35)	8 (40)	7 (35)	7 (35)	0.987c
	Middle	9 (45)	8 (40)	9 (45)	7 (35)	
	High	4 (20)	4 (20)	4 (20)	6 (30)	
Education	Middle (12–15 years of education)	14 (70)	14 (70)	13 (65)	15 (75)	0.986b
	High (16 or more years of education)	6 (30)	6 (30)	6 (35)	5 (20)	
Employment	No	8 (40)	8 (45)	7 (35)	5 (25)	0.659b
	Yes	12 (60)	11 (55)	13 (65)	15 (75)	
Marital status	Single	11 (55)	14 (70)	13 (65)	14 (70)	0.279c
	Married	9 (45)	3 (15)	6 (30)	4 (20)	
	Divorced	0	3 (15)	1 (5)	2 (10)	

**Group I**: No cannabis use (Control); **Group II**: Cannabis use for 1–2 years; **Group III**: Cannabis use for 5–6 years; **Group IV**: Cannabis use for 9–10 years

a; Kruskal Wallis test, b; Chi square test, c; Fisher Exact test, *p is significant at < 0.05

Table 2 shows that mental flexibility, programming, and inhibitory control were significantly different across the study groups (*p* = 0.022, 0.009, and 0.000, respectively), where worse performance exist with increased duration of cannabis use. Other FAB domains showed no statistically significant differences. Increasing duration of Cannabis use was also associated with increasing percentage of affected frontal lobe assessment battery (*p* = 0.001).

**Table 2:** FAB (total and domain-specific scores) between all study groups (Cannabis users and controls) (*n* = 80)

FAB domains	Assessment Criteria	Study groups, n (%)				Test value	*p*-value
		Group I (*n* = 20)	Group II (*n* = 20)	Group III (*n* = 20)	Group IV (*n* = 20)		
**Similarity** (Conceptualization)	One correct	0	0	2 (10.0)	2 (10.0)	8.43 ^a^	0.115
	Two correct	0	2 (10.0)	2 (10.0)	4 (20.0)		
	Three correct	20 (100.0)	18 (90.0)	16 (80.0)	14 (70.0)		
**Lexical Fluency** (Mental Flexibility)	Less than 3 words	0	0	2 (10.0)	2 (10.0)	14.70 ^a^	0.022 *
	3–5 words	0	0	2 (10.0)	2 (10.0)		
	6–9 words	0	2 (10.0)	4 (20.0)	4 (20.0)		
	More than 9 words	20 (100.0)	18	12 (60.0)	12 (60.0)		
**Motor Series Test** (Programming)	3–5 correct consecutive series alone	1 (5.0)	2 (10.0)	6 (30.0)	9 (45.0)	11.27 ^a^	0.009 *
	6 correct consecutive series alone	19 (95.0)	18 (90.0)	14 (70.0)	11 (55.0)		
**Conflicting instructions** (Sensitivity to Interference)	More than 2 errors	0	1 (5.0)	0	0	4.12 ^a^	0.775
	1–2 errors	9 (45.0)	10 (50.0)	9 (45.0)	12 (60.0)		
	No errors	11 (55.0)	9 (45.0)	11 (55.0)	8 (40.0)		
**Go-No Go** (Inhibitory Control)	More than 2 errors	0	1 (5.0)	0	0	19.47 ^a^	< 0.001 *
	1–2 errors	2 (10.0)	9 (45.0)	11 (55.0)	14 (70.0)		
	No errors	18 (90.0)	10 (50.0)	9 (45.0)	6 (30.0)		
**Prehension behavior** (Environmental Autonomy)	Take hands without hesitation	0	1 (5.0)	2 (10.0)	2 (10.0)	4.37 ^a^	0.645
	Hesitate or ask what to do	5 (25.0)	8 (40.0)	7 (35.0)	8 (40.0)		
	Not take the examiner’s hands	15 (75.0)	11 (55.0)	11 (55.0)	10 (50.0)		
**Total FAB Score**	Not affected	15 (75.0)	7 (35.0)	7 (35.0)	3 (15.0)	15.83 ^b^	0.001 *
	Affected	5 (25.0)	13 (65.0)	13 (65.0)	17 (85.0)		

**Group I**: No cannabis use (Control); **Group II**: Cannabis use for 1–2 years; **Group III**: Cannabis use for 5–6 years; **Group IV**: Cannabis use for 9–10 years

^a^. Fisher-Freeman-Halton Exact Test. ^b^. Chi-square test

*. Statistically significant at *p* < 0.05

- Variables have been treated as separate categories

**Fig. 1 Total Frontal lobe Assessment Battery scores between all study groups:**
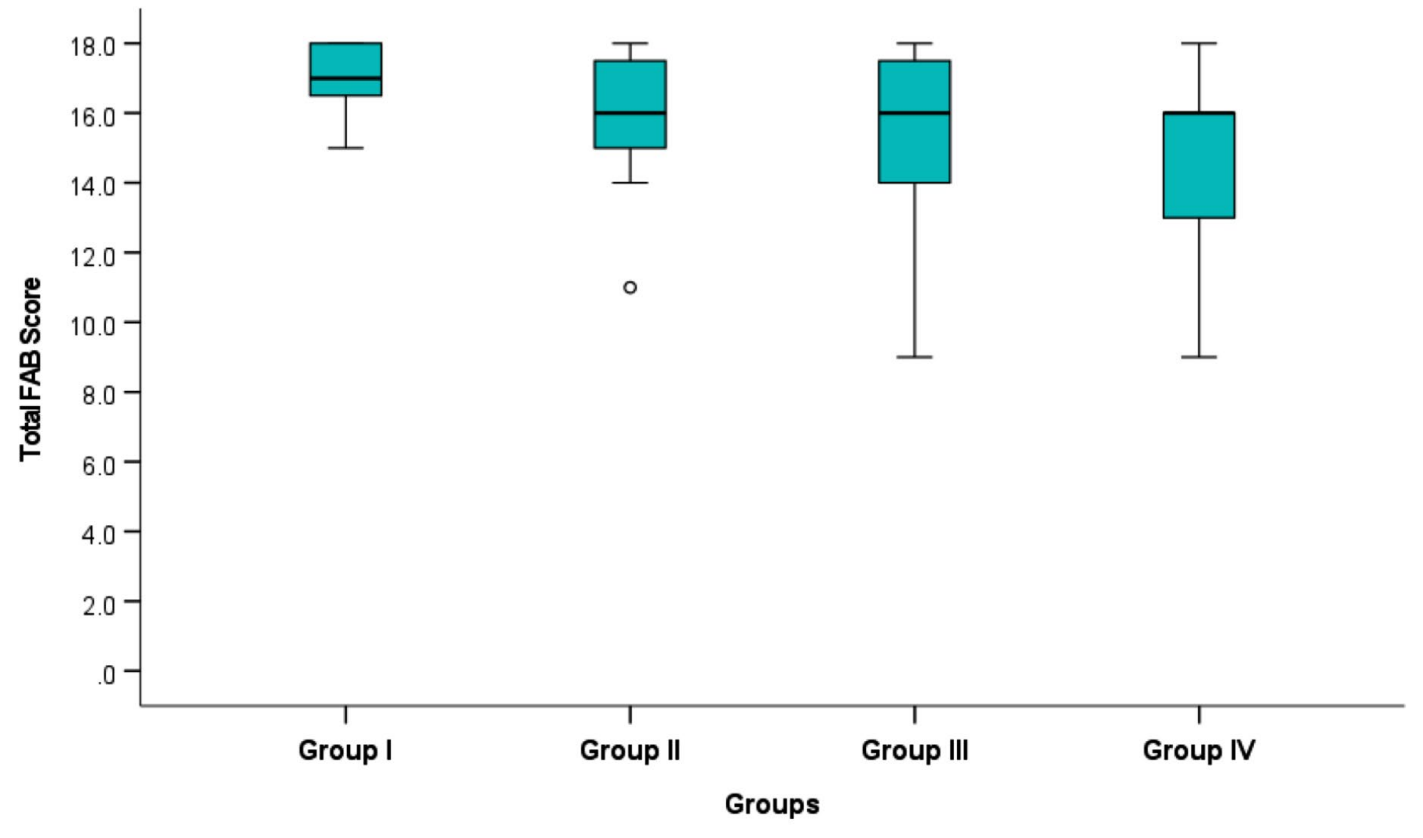
.

Table 3 shows that the total FAB score was significantly different across the study groups (*p* = 0.004). Post-hoc testing revealed that the significant difference existed only between Cannabis non-users (group I) and the longest duration of Cannabis use (group IV), (*p* = 0.003) (Fig. 1).

**Table 3:** Differences in total FAB score between all study groups (Cannabis users and controls) (*n* = 80)

	Total FAB score Median (IQR)	Test value ^a^	*df*	*p*-value	Pairwise Comparisons Test value (*p*-value ^b^)			
					Group I	Group II	Group III	Group IV
Group I	17.0 (1.5)	13.10	3	0.004 *		15.80 (0.166)	17.65 (0.084)	25.25 (0.003*)
Group II	16.0 (2.5)				15.80 (0.166)		1.85 (1.00)	9.45 (1.00)
Group III	16.0 (3.5)				17.65 (0.084)	1.85 (1.00)		7.60 (1.00)
Group IV	16.0 (3.0)				25.25 (0.003*)	9.45 (1.00)	7.60 (1.00)	

**Group I**: No cannabis use (Control); **Group II**: Cannabis use for 1–2 years; **Group III**: Cannabis use for 5–6 years; **Group IV**: Cannabis use for 9–10 years; **FAB**: Frontal lobe assessment battery; **IQR**: Interquartile range; **df**: degree of freedom

^a^. Independent-Samples Kruskal-Wallis Test

^b^. Significance values have been adjusted by the Bonferroni correction for multiple tests

*. Statistically significant at *p* < 0.05

**Fig. 2 Total General Assessment of Function (GAF) scores among groups of Cannabis use:**
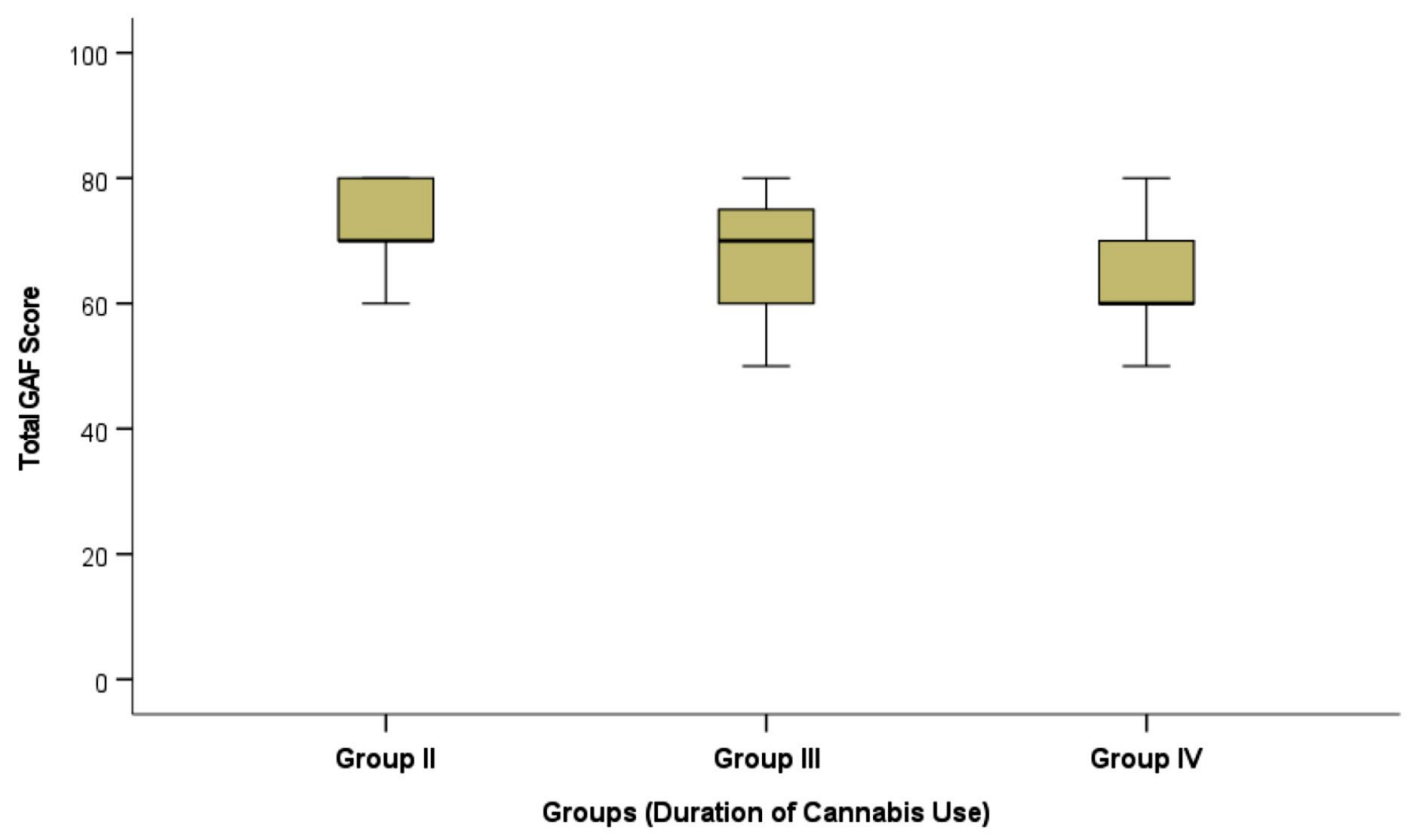
.

Likewise, the total GAF score was significantly different across the Cannabis users; groups II-IV (*p* = 0.006), where prolonged duration of cannabis use is associated with lower scores of GAF and more impairment in social, occupational, and psychological functioning (Table 4). Post-hoc testing revealed that the significant difference existed only between the shortest (group II) and the longest duration (group IV) of Cannabis use, (*p* = 0.009) (Fig. 2).

**Table 4:** Differences in total GAF score between groups of Cannabis use (*n* = 60)

	Total GAF score Median (IQR)	Test value ^a^	*df*	*p*-value	Pairwise Comparisons Test value (*p*-value ^b^)		
					Group II	Group III	Group IV
Group II	70.0 (10.0)	10.14	2	0.006 *		9.83 (0.376)	16.73 (0.009 *)
Group III	70.0 (15.0)				9.83 (0.376)		6.90 (1.00)
Group IV	60.0 (10.0)				16.73 (0.009 *)	6.90 (1.00)	

**Group II**: Cannabis use for 1–2 years; **Group III**: Cannabis use for 5–6 years; **Group IV**: Cannabis use for 9–10 years; **GAF**: Global assessment of function; **IQR**: Interquartile range; **df**: degree of freedom

^a^. Independent-Samples Kruskal-Wallis Test

^b^. Significance values have been adjusted by the Bonferroni correction for multiple tests

*. Statistically significant at *p* < 0.05

Table 5 shows that there were no statistically significant correlations between the duration of Cannabis use and neither FAB nor GAF total scores, adjusted for the age-of-onset (*p* = 0.984, and 0.138, respectively). However, the age-of-onset showed significant positive medium correlation with either FAB or GAF total scores (*r* = 0.420, *p* < 0.001; *r* = 0.511, *p* < 0.001, respectively), and significant negative strong correlation with the duration of Cannabis use (*r* =-0.770, *p* < 0.001), adjusted for participants’ age. Furthermore, the FAB and GAF total scores showed significant positive and medium correlation among Cannabis users (*r* = 0.504, *p* < 0.001), adjusted for the age-of-onset and duration of use. All FAB domains, except for environmental autonomy, were also significantly and positively correlated with the total GAF score), adjusted for the age-of-onset and duration of use (Table 6).

**Table 5:** Correlations of total GAF and FAB scores with the duration of Cannabis use and age of onset among Cannabis users (*n* = 60)

	Duration of Cannabis Use			Age of Onset (years)	
	*r* ^a^	*p*-value	*r*^b^(95% CI)		*p*-value
Total FAB score	0.003 (-0.184, 0.215)	0.984	0.420 (0.169, 0.618)		< 0.001 *
Total GAF score	-0.196 (-0.433, 0.065)	0.138	0.511 (0.326, 0.684)		< 0.001 *
Duration of Cannabis Use	1.000	**-**	-0.770 (-0.856, -0.665)		< 0.001*

**FAB**: Frontal lobe assessment battery; **GAF**: Global assessment of function; ***r***: Partial correlation coefficient; **CI**: Confidence interval

^a^. adjusted for age of onset

^b^. adjusted for age

*. Statistically significant at *p* < 0.05

**Table 6:** Correlations between GAF and FAB (total and domain-specific scores) among Cannabis users (*n* = 60)

	Total GAF score	
	*r*^a^(95% CI)	*p*-value
**FAB Domains**:		
***Similarity*** *(Conceptualization)*	0.397 (0.238, 0.546)	0.002 *
***Lexical Fluency*** *(Mental Flexibility)*	0.620 (0.422, 0.762)	< 0.001 *
***Motor Series Test*** *(Programming)*	0.529 (0.115, 0.277)	< 0.001 *
***Conflicting instructions*** *(Sensitivity to Interference)*	0.274 (0.002, 0.507)	0.037 *
***Go-No Go*** *(Inhibitory Control)*	0.302 (0.032, 0.531)	0.021 *
***Prehension behavior*** *(Environmental Autonomy)*	0.017 (-0.242, 0.250)	0.901
**Total FAB score**	0.504 (0.278, 0.657)	< 0.001 *

**FAB**: Frontal lobe assessment battery; **GAF**: Global assessment of function; ***r***: Partial correlation coefficient; **CI**: Confidence interval

^a^. adjusted for age of onset and duration of Cannabis use

*. Statistically significant at *p* < 0.05

Table 7 shows that the association between the duration of Cannabis use (groups) and either FAB or GAF total scores were not significant after adjusting for the confounding effect of the age-of-onset and the interaction between age-of-onset and groups. The only significant predictor for the total FAB and GAF scores was the age-of-onset, where every one-year increase in the age-of-onset was significantly associated with 0.55 and 3.53 unit increases in the median total FAB and GAF scores, respectively (*p* = 0.043, and 0.019).

**Table 7:** Regression analysis to evaluate the adjusted association between the duration of Cannabis use (groups) and total FAB and GAF scores among Cannabis users (*n* = 60)

	Parameter Estimates (q = 0.5)					
	Coefficient	SE	t value	df	*p*-value	95% CI
**Total FAB score**						
*(Intercept)*	6.86	4.34	1.58	56	0.120	-1.83, 15.55
*Groups*	1.86	1.98	0.94	56	0.353	-2.12, 5.83
*Age of onset*	**0.55**	**0.27**	**2.07**	**56**	0.043 *	0.02, 1.08
Groups * Age of onset	-0.12	0.11	-1.08	56	0.285	-0.35, 0.108
**Total GAF score**						
*(Intercept)*	4.71	23.82	0.20	56	0.844	-43.01, 52.42
*Groups*	21.77	10.90	2.00	56	0.051	-0.06, 43.59
*Age of onset*	**3.53**	**1.46**	**2.42**	**56**	0.019 *	0.60, 6.46
Groups * Age of onset	-1.18	0.62	-1.89	56	0.064	-2.42, 0.07

**Groups: II**: Cannabis use for 1–2 years; **III**: Cannabis use for 5–6 years; **IV**: Cannabis use for 9–10 years; **FAB**: Frontal lobe assessment battery; **GAF**: Global assessment of function; **SE**: Standard error; **df**: degree of freedom; **CI**: Confidence interval

## Discussion

This study focused on residual neurocognitive effects of cannabis use, and its association with the person’s general level of functioning (occupational and psychosocial functioning) assessed on the General Assessment of Function (GAF) scale. In our study, frontal lobe neurocognitive functioning of cannabis users (abstinent for one month to 3 months) and controls was evaluated by using the Frontal lobe Assessment Battery (FAB).

We included the sociodemographic data of our sample in Table (1). In Table (2) when FAB subtests were analyzed, there was statistically significant impairment in cannabis users’ groups when compared to control group in mental flexibility, motor series test (programming), inhibitory control. Fontes et al. (2011) also found that cannabis users performed worse only in the Motor Programming subtest, this may be explained by the difference in control selection, as the other study had control group of cannabis naïve or abstinent for more than three months which may have showed residual cognitive dysfunction.

Al-Hakeem et al. (2020); Cunha et al. (2010), both confirmed the negative effect of cannabis use on FAB subscales stating the negative impact on (abstract reasoning, motor programming, and cognitive flexibility), and (abstract reasoning, motor programming, and inhibitory control) respectively.

As shown in Table (3) and Fig. (1) the longer duration of cannabis usage was associated with worse frontal lobe battery scores. Our findings are consistent with Stypulkowski & Thayer, (2022) who found that duration of cannabis use has a crucial role in cognitive function, with affection in long-term users compared to nonusers or short-term users.

We performed our tools to participants who were abstinence for a period between 1 and 3 months, although their cognitive scores were below those of control group.

Our finding is consistent with Lovell et al. (2018) that long term cannabis use significantly affects cognitive performance negatively. (Lorenzetti et al. 2021) also stated that even after abstinence, verbal learning and visuospatial skills were below expected.

Research revealed conflicting results according to cognitive functions after abstinence, suggesting that factors as age of onset (Albaugh et al., 2023), frequency of use, duration of abstinence prior to assessment, and cumulative exposure to cannabis impact cognitive functions. According to (Broyd et al. 2016; Ganzer et al. 2016); residual cognitive impairments are linked to the duration and quantity of cannabis use, regardless the” age of onset”.

In another systematic review (Lovell et al., 2020) found that long-term cannabis use was associated with worse learning and memory, and global cognitive functioning than controls, all of which persisted even after 25 days of abstinence.

Another meta-analysis demonstrated that verbal learning improved within 7 to 28 days of sustained abstinence. However, the duration of use was inversely related to longer periods of abstinence to this improvement to occur, undermining a confident inference that abstinence alone has direct benefits to verbal learning and memory (Krzyzanowski & Purdon, 2020).

(Debenham et al. 2021) stated that no recovery with medium-term abstinence in users for more than 7·5 years, suggesting that longer period of abstinence is required for long-term user, indicating that resilience is related to the duration of use. In “a threshold theory of harm”, neurotoxicity was affected by the frequency of use, and the abstinence duration for recovery depends on duration of use and age of onset as deterioration in neuroplasticity make recovery more difficult with prolonged use (Batalla et al. 2013; Debenham et al. 2021).

As shown in Table (3), Fig. (2), The global assessment of function (GAF) in abstinent cannabis users’ groups showed significant difference indicating that prolonged duration of cannabis use was associated with lower occupational and psychosocial (family and other) functioning levels.

Our results are in concordance with the results of (Crean et al. 2011) who mentioned the relation between cognitive affection secondary to cannabis usage and that it can affect their ability to learn, make decisions, behave, or even feel properly.

We have assessed cannabis use duration, its associated neurocognitive impairment, and its correlation with global assessment of occupational and psychosocial functioning status of participants. Global Assessment of Functioning scores (that indicated a person’s level of occupational and psychosocial functioning) were correlated with the neurocognitive performance assessed with the neuropsychological battery used to evaluate frontal lobe functions of an individual, showing the crucial role of cognitive performances of individuals in their overall functioning level in life, with more significant correlation particularly with long-term cannabis use and earlier age of onset. A systematic review was conducted that searched into 13 studies testing chronic cannabis users compared with controls and it showed that impairments of frontal lobe functions secondary to cannabis use were associated with decrease in their ability to communicate and deal in social and occupational tasks making users more susceptible to adverse life events and to the development of new psychiatric symptoms (Figueiredo et al. 2020; Sorkhou et al. 2021).

As earlier age of onset of cannabis use is known to be associated with more susceptibility for cannabis use disorder and associated with longer duration with cannabis use (Curran et al. 2023), in Table (5) and (6) correlation between neurocognitive frontal lobe functions (FAB) and the general level of occupational and psychosocial functioning (GAF) were studied regarding duration of cannabis use and age of onset of cannabis both showing that earlier age of onset was correlated with more affection of both FAB and GAF. This conforms with results of (Scheyer et al. 2023) who found that cannabis use during vulnerability window (e.g., adolescence) affects the neurodevelopment and behavior by changing synaptic and dendritic structure, resulting in affection in social, and occupational life.

## Conclusion

This study indicates that individuals with CUD are at a higher risk of exhibiting neurocognitive deficits that develop as a consequence of prolonged cannabis use. These effects linger even after a period of abstinence, worsen with prolonged duration, and are associated with concurrent overall affection of individual functioning level in the occupational, family, and other social aspects of his life.

Educating our communities about those intertwining negative consequences associated with cannabis use and CUD is crucial to obtain a shift in the public understanding of the current underrated perceived harm resulting from prolonged cannabis use.

## Limitations, strengths, and future directions

Our study builds upon existing literature in a multivariate framework that can help in better understanding the long-term neurocognitive effects of chronic cannabis use on individual’s occupational and psychosocial functioning, even after abstinence. Most of the previous research studies that explored the neurocognitive effects of cannabis, either acute or chronic, included mainly adolescents. We included adults in this study. Erikson described this period as the period of intimacy vs. isolation (young adulthood, 18–35 years with a primary task of forming stable long-lasting relationships) and generativity vs. stagnation (middle adulthood, 35–55 where being productive is the key to a balanced life) (Malone et al., 2016).

Negative effects on individual level of functioning, can have implications on both the personal and national levels.

In this research, we also tried to overcome some methodological limitations in past research studies as heterogeneity of the population under study, through applying a participant selection that puts in consideration possible confounding factors (e.g., including only males, controls that were relatives to cases, and excluding any comorbid medical or psychiatric conditions).

A number of limitations exists in our study. The relatively small sample size, being taken form one addiction treatment hospital along with the cross-sectional study design make it difficult to draw any definite inferences on the causal relationship between the lingering neurocognitive effects of cannabis after a period of abstinence and the current general level of occupational and psychosocial functioning in cannabis use disorder patients. We included only males in this study, this hinders the broad generalization of this study results on both sexes.

We grouped our sample post hoc based on the duration of use of participants presented to the clinic at the time of participant recruitment to the study. Future studies should consider including a representative sample covering the whole period of a 10 years’ time after abstinence without interruption.

We did not perform any quality-of-life assessments on our sample, this would have enriched our results regarding data covering life affection of users. Lack of baseline data (prior to cannabis use in cases and inclusion in the study in controls) of the participants’ neurocognitive functions, IQ levels and their general level of functioning are considered conservable limitations as these obscures the current neuropsychological and functional state results. Future studies should consider longitudinal design and take these points into consideration. This is a case-control study with a retrospective design that relied on self-reported abstinence data, thus subjecting the results to the possibility of recall biases, particularly as the cannabis use disorder cases that participated were treatment seekers. Further research that includes both treatment-seeking and non-treatment seeking cases are needed to overcome this potential bias.

### Electronic supplementary material

Below is the link to the electronic supplementary material.

Supplementary Material 1

Supplementary Material 2

Supplementary Material 3

Supplementary Material 4

Supplementary Material 5

## Data Availability

Available from the corresponding author upon responsible request.

## Abbreviations

DSM-V: Diagnostic and Statistical Manual of Mental Disorders, Fifth Edition
FAB: Frontal Assessment Battery
GAF: Global Assessment of Functioning
SCID-V - RV: Structured Clinical Interview for DSM-V Research Version
