# Supplementary material for: Assessment of frontal lobe functions in a sample of male cannabis users currently in abstinence: correlations with duration of use and their functional outcomes
Source: J Cannabis Res. 2024 Aug 21;6:34. doi: 10.1186/s42238-024-00244-w (PMC11337584; doi:10.1186/s42238-024-00244-w)
Supplement: Supplementary file 4 — Supplementary Material 4 [file 42238_2024_244_MOESM4_ESM.docx]

**Supplement Histogram 2 (S2).**


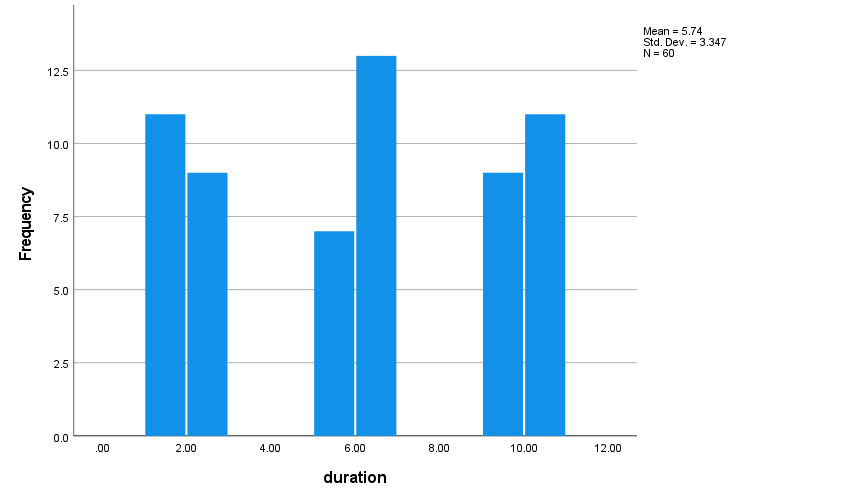


**(S2).** A histogram describing the distribution **of cannabis use duration** across the whole sample.
