# Supplementary material for: Assessment of frontal lobe functions in a sample of male cannabis users currently in abstinence: correlations with duration of use and their functional outcomes
Source: J Cannabis Res. 2024 Aug 21;6:34. doi: 10.1186/s42238-024-00244-w (PMC11337584; doi:10.1186/s42238-024-00244-w)
Supplement: Supplementary file 1 — Supplementary Material 1 [file 42238_2024_244_MOESM1_ESM.pdf]

Suez Canal University
Faculty of Medicine
Research Ethics Committee

Date: 29/04/2024

Dr: ***El Shimaa Ibraheem Mohammed Tag Eldeen***

Department of: Neurology and Psychiatry, Faculty of Medicine, Suez Canal University, Ismailia, Egypt

Reference: Research 4350#

Thesis Title: ***Assessment of Cognitive Function Abnormalities in A Sample of Regular Cannabis users.***

Dear Dr,

Thank you for your letter in (29/04/2024) to ask for the ethical clearance previously given for the above mentioned protocol.

We are pleased to confirm that the above mentioned protocol of thesis was for the partial fulfillment of *Doctorate degree* in *Psychiatry* for: *El Shimaa Ibraheem Mohammed Tag Eldeen,* under the supervision of Prof. Magda Taha Kamal Eldeen Fahmy, *Prof. Khaled Abd Elmoez Mohamed, and Dr. Omneya Youssef Muhammad Ibrahim.*

The proposal of the above mentioned protocol was reviewed by the full board Committee at its meeting on (23/11/2020) and **the ethical clearance was given for that proposal.**

Yours sincerely,

Prof. Amani El-Baz
Co-Chair of Research Ethics Committee
Faculty of Medicine
Suez Canal University

Amani El Baz
30/4/2024

جامعة قناة السويس
كلية الطب البشرى
لجنة اخلاقيات البحث العلمى

Mail address: Research Ethics Committee, Faculty of Medicine, Suez Canal Universit
Ring Road, Ismailia, Egypt. **P.O. Box:** 41522 **E-mail:** rec@med.suez.edu.eg
Phone:064-3200789
