# Supplementary material for: Assessment of frontal lobe functions in a sample of male cannabis users currently in abstinence: correlations with duration of use and their functional outcomes
Source: J Cannabis Res. 2024 Aug 21;6:34. doi: 10.1186/s42238-024-00244-w (PMC11337584; doi:10.1186/s42238-024-00244-w)
Supplement: Supplementary file 2 — Supplementary Material 2 [file 42238_2024_244_MOESM2_ESM.docx]

We wish to thank the Editor and Reviewers very much for their remarkable efforts and are sincerely grateful for their valuable review of our manuscript. The careful and thoughtful reviews and insightful comments and suggestions kindly outlined, led to the notable improvement of the quality of our manuscript and we believe and hope the revised version of the manuscript would lead to a better impact on the readers.

We have addressed each of the further concerns and comments below, to the best we could, on a point-by- point basis and made changes to the manuscript accordingly. We highlighted the changes made in grey in the main manuscript file.

Reviewer #2 comments:

Reviewer #2: The authors have done a good job of addressing the points raised by myself and the other reviewers. Subsequently, the quality of the manuscript has been greatly improved. The portion of the manuscript where the revisions have made the most impact is the Methods section. I think these findings will be a valuable addition to the cannabis research landscape. However, I do have some minor points that I think should be addressed, which I will go over by section.

Author Response:

We wish to thank Reviewer #2 for the valuable positive comment and the opportunity of revising the work further.

OVERALL COMMENTS:

- The authors refer to the GAF as measuring "functioning" or "global functioning." Given that they also often refer to "frontal lobe functioning," I find it a bit confusing as to what "global functioning" is referring to. This first comes up in the Abstract, but continues throughout the manuscript. I think it would benefit to add in a short description of what kind of functioning the GAF is referring to (occupational? daily life?) in the abstract and in the Introduction when the measures are first introduced, and to carefully edit the manuscript to make sure that when both the FAB and GAF measures are being referred to, it is clear which kind of "functioning" is being talked about.

Author Response:

Thank you so much for this suggestion for a better clarity of the manuscript. We acknowledge that unnecessary perplex might arise from not delineating clearly what we mean when we refer to the “function” within the 2 contexts. We agree with Reviewer #2 assessment and have added a short description of what we mean by “functioning” within each mention as per recommended, throughout the manuscript.

BACKGROUND:

- I feel that the comment about "worker productivity" being impacted by cannabis use still has not been justified sufficiently. The authors cite an article that shows that cannabis can impair cognitive function, but provide no citation or evidence to then link changes in cognitive function to worker productivity behaviors. I understand it is probably "common sense" that cognitive functioning would have a effect on worker productivity, but if the authors cannot find a citation to back up this claim (or at least an article that directly finds that cannabis use effects worker productivity), this point about "worker productivity" should be taken out of the manuscript.

Author Response:

We thank Reviewer #2 for this salient comment and acknowledge the vagueness of the previous mention of “worker productivity” in the referenced comment. We have rewritten this explanation and updated the reference accordingly.

- "Blunted stress reactivity ... can hold both resiliency and vulnerability" --> how is blunted stress reactivity associated with both resiliency and vulnerability? Please include a citation for this.

Author Response:

We appreciate the thorough revision and suggesting revision of this point in the “Background” section that we totally agree that it was unclear. We have rewritten the paragraph where we have mentioned the effect of cannabis use on the stress response and cortisol release and updated the references accordingly.

- I think the manuscript would benefit from a brief paragraph at the end of the Background/Introduction with an overview of what the main research questions are and how they were tested, to increase clarity for the reader.

Author Response:

We have added a paragraph that included our research aim, questions and hypotheses at the end of the “Background” section as per recommended.

METHODS:

- Thank you for including details about the power analysis that was run to justify the sample sizes. However, I am wondering where the expected effect size of 0.4 comes from? Is this based on previous literature that has seen effect sizes of that approximate magnitude?

Author Response:

We would like to thank Reviewer #2 for this encouraging positive comment. Upon conducting our search to gather background information about our research topic and questions, we found previous literature that suggested the presence of an association between duration of cannabis use and frontal lobe dysfunction; however, we did not find an estimate for the effect size of this association in the literature. Therefore, we relied on the interpretation of Cohen’s f2 Statistic – which suggest that the values of 0.10, 0.25, and 0.40 represent small, medium, and large effect sizes, respectively – and also upon expert opinion about the approximate magnitude of the effect size.

- While the authors explained the reason for the group divisions (1-2 years, 5-6 years, and 9-10 years) in their responses to the reviewers, this information needs to be explicitly added in the Methods section of the manuscript. I also think that since these group divisions were chosen post hoc based on the duration of use presented in their sample, and not based on any prior hypothesis or meaningful expected difference between the groups in previous literature, this should be discussed as a limitation in the Discussion.

Author Response:

We would like to thank Reviewer #2 for this suggestion. We have added this information to the “Methods” section, and we have added the part related to the sample division as a limitation in the “Discussion” section.

RESULTS:

- Given the importance of age of onset throughout the manuscript, I think it would be useful to include a histogram that shows the distribution of age of onset throughout the whole sample (this can be included as a Supplement, and it is probably alright if it is not included in the main text).

Author Response:

We would like to thank Reviewer #2 for this valuable suggestion. We have added a histogram showing the distribution of age of onset throughout the whole sample as “Supplement 1.”, and we referred to it in the “Results” section of the revised version of the manuscript. We have referred to this supplement in the “Results” section.

- Similarly, I would like to see a histogram describing the distribution of cannabis use duration across the sample (continuously, not grouped together), if this data is available. This can also be included in the Supplement if needed.

Author Response:

We have added a histogram that showing the description of the distribution of cannabis use duration across the sample, presented continuously as per suggested. It is referred to as “Supplement 2.” We also have referred to this supplement in the “Results” section.

- I would like to see a more comprehensive description of the sub-tasks that are being compared in Table 2. For example, since I am not familiar with the assessments, "3-5 correct consecutive series" does not mean anything to me. I think the authors should describe what is being measured and what the scores on each sub-test mean in the Methods section.

Author Response:

We would like to thank Reviewer #2 so much for this salient observation. We acknowledge that it can be ambiguous for the reader to read table 2 without prior suitable description of the tool. We have added a detailed description of the tool in the “Methods” section.

- Relatedly, I am wondering where the levels of the assessment score factors come from? E.g. why are "less than 3 words," "3-5 words," "more than 9 words," treated as 3 separate categories rather than a continuous variable (also, where did the division between these categories come from? Is there a meaningful reason, or were they arbitrarily selected)? This can be applied to all of the sub-tests in this table. If the scores are grouped this way because it is the way that they are intended to be analyzed by the assessment tool, this needs to be clearly

described in either the Methods or the caption of the table; if not, the choice to divide scores up in this way needs to be explained and justified.

Author Response:

We would like to thank Reviewer #2 for this meticulous notice. We have followed the analysis of the assessment tool itself. We think this is the way they intended it to be analyzed. We have added that notice as a caption of the table as recommended.

DISCUSSION:

- Some paragraphs in the Discussion would be better suited in the Background as they more give examples of ways cannabis can impact cognition/brain function without referring back to the Results of the manuscript, e.g. the last 2 paragraphs referring to fMRI studies and cortical thickness studies.

Author Response:

We would like to thank Reviewer #2 for this comment. We totally agree that these 2 paragraphs did not belong to the “Discussion” and have removed them accordingly form that section.

- "We included adults in this study as this is the period of the highest functional capacity in the person's life." --> I am not really sure what this sentence is referring to ("functioning" is vague), and either way it would need a citation.

Author Response:

Thank you so much for this important remark. Indeed, we agree upon the importance of clarifying this point further. We have rewritten this part and provided a citation. We have also included a paragraph briefly mentioning some of the frontal lobe functions that are salient for the person to accomplish his daily functions so that when introduce previous research discussing cannabis effects, it would be more comprehensible for the reader to digest what we mean by impairment in frontal lobe functions.

**Much obliged**

Omneya Ibrahim

(The corresponding author)
